# Detection and discrimination of influenza B Victoria lineage deletion variant viruses by real-time RT-PCR

**DOI:** 10.2807/1560-7917.ES.2020.25.41.1900652

**Published:** 2020-10-15

**Authors:** Bo Shu, Marie K Kirby, Christine Warnes, Wendy M Sessions, William G Davis, Ji Liu, Malania M Wilson, Stephen Lindstrom, David E Wentworth, John R Barnes

**Affiliations:** 1 Virology, Surveillance and Diagnostic Branch, Influenza Division, Centers for Disease Control and Prevention, Atlanta, United States; 2 Battelle Memorial Institute, Atlanta, United States; 3 Chickasaw Nation Industries, Inc., Atlanta, United States; 4 Respiratory Virus Branch, Division of Viral Diseases, Centers for Disease Control and Prevention, Atlanta, United States

**Keywords:** B/Victoria lineage, deletion variant virus, real-time RT-PCR

## Abstract

**Background:**

During the 2016/17 influenza season, influenza B/VIC lineage variant viruses emerged with two (K_162_N_163_) or three (K_162_N_163_D_164_) amino acid (aa) deletions in the haemagglutinin (HA) protein. There are currently five antigenically distinct HA proteins expressed by co-circulating influenza B viruses: B/YAM, B/VIC V1A (no deletion), B/VIC V1A-2DEL (2 aa deletion) and two antigenically distinguishable groups of B/VIC V1A-3DEL (3 aa deletion). The prevalence of these viruses differs across geographical regions, making it critical to have a sensitive, rapid diagnostic assay that detects and distinguishes these influenza B variant viruses during surveillance.

**Aim:**

Our objective was to develop a real-time RT-PCR (rRT-PCR) assay for detection and discrimination of influenza B/VIC lineage variant viruses.

**Methods:**

We designed a diagnostic assay with one pair of conserved primers and three probes specific to each genetic group. We used propagated influenza B/VIC variant viruses and clinical specimens to assess assay performance.

**Results:**

This rRT-PCR assay detects and distinguishes the influenza B/VIC V1A, B/VIC V1A-2DEL, and B/VIC V1A-3DEL variant viruses, with no cross-reactivity. This assay can be run as a multiplex reaction, allowing for increased testing efficiency and reduced cost.

**Conclusion:**

Coupling this assay with the Centers for Disease Control and Prevention’s Human Influenza Virus Real-Time RT-PCR Diagnostic Panel Influenza B Lineage Genotyping Kit results in rapid detection and characterisation of circulating influenza B viruses. Detailed surveillance information on these distinct influenza B variant viruses will provide insight into their prevalence and geographical distribution and could aid in vaccine recommendations.

## Introduction

Influenza B viruses co-circulate with influenza A strains during the annual influenza season, contributing to overall mortality and morbidity of influenza epidemics. In the 1980s, influenza B viruses evolved into two genetically and antigenically distinct lineages represented by B/Victoria/2/1987 (VIC) and B/Yamagata/16/1988 (YAM) that co-circulate in human hosts during influenza seasons worldwide [1-5]. During the 2016/17 influenza season, the United States (US) Centers for Disease Control and Prevention (CDC) detected influenza B/VIC viruses in the US that were antigenically distinct from the World Health Organization (WHO)-recommended vaccine virus, B/Brisbane/60/2008 (VIC) [6]. Sequencing analysis confirmed that these viruses have a deletion of 6 nucleotides (nt) in the haemagglutinin (HA) gene resulting in a 2 amino acid (aa) deletion at positions 162 and 163 (corresponding to nt positions 529–534) [6]. This B Victoria lineage 2 aa deletion virus (V1A-2DEL; K_162_N_163_ deletion) has since spread and been detected worldwide. Two B/VIC lineage variants with a deletion of 3 aa (V1A-3DEL) at aa positions 162–164 (K_162_N_163_D_164,_ i.e. nt positions 529–537) emerged shortly after via parallel evolution. The V1A-3DEL viruses have since been detected in Asia, Africa, Europe and America [7]. There are currently five genetically distinct HA genes that yield five antigenically distinct influenza B viruses that are co-circulating: B/YAM, B/VIC V1A, B/VIC V1A-2DEL and two groups of B/VIC V1A-3DEL (Figure) [8].

**Figure:**
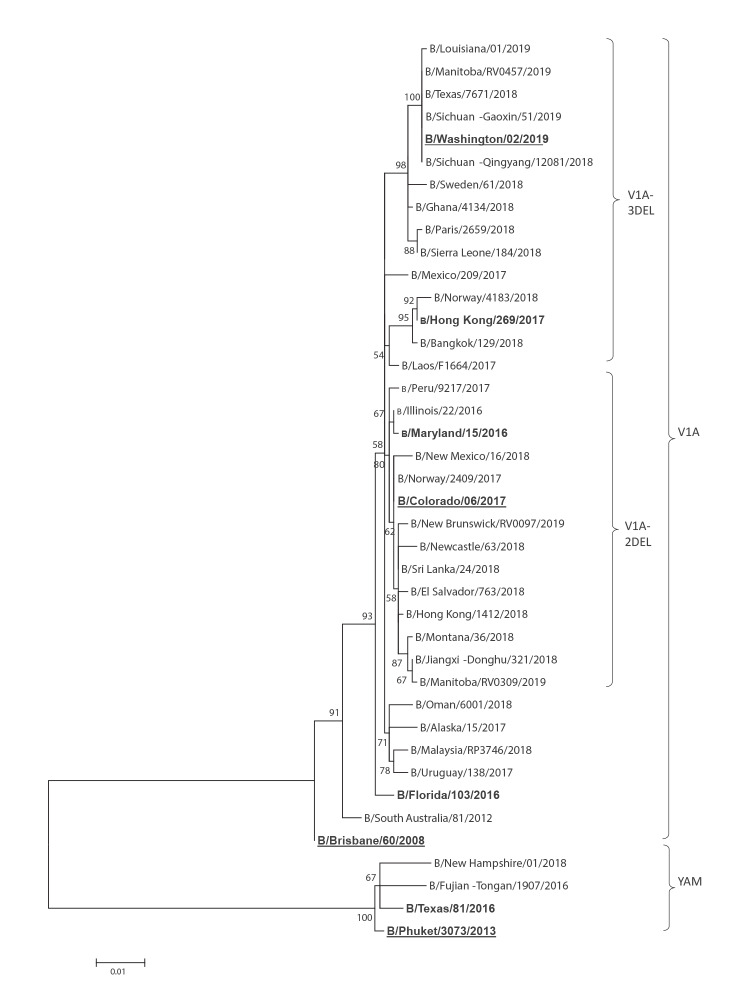
Evolutionary relationships among influenza B Victoria and Yamagata haemagglutinin genes

HA: haemagglutinin.

HA (coding sequence) phylogenetic tree with representative viruses of the genetic groups of B/Victoria and B/Yamagata lineages of related viruses; V1A, V1A-2DEL, V1A-3DEL and YAM are indicated by the bars on the right. The viruses evaluated in this study are shown in bold and influenza B vaccine viruses are underlined. HA sequences used in this analysis are detailed in Supplementary Table S1. Phylogenetic analysis was performed using Molecular Evolutionary Genetics Analysis software (MEGA, version 7.0). The evolutionary history was inferred using the maximum likelihood method. The evolutionary distances were computed using the Tamura–Nei method. The bootstrap value (1,000 replicates) of the HA gene is shown next to the branches.

The most effective method for prevention and control of influenza infection is vaccination [3,4,9]. Licensed seasonal vaccines are updated annually and the WHO makes recommendations on the composition of influenza virus vaccines on the basis of surveillance, laboratory and clinical observations [3]. This process occurs twice a year, in February for the northern hemisphere and in September for the southern hemisphere. It is important to identify the most prevalent influenza A subtype and B lineage viruses through influenza surveillance for influenza vaccine selection, especially for people who may not have been exposed to a subtype/lineage of influenza virus [10]. Identifying the optimal viruses to include in the next season’s influenza vaccines is a significant challenge for the influenza surveillance network known as the Global Influenza Surveillance and Response System (GISRS). A diagnostic assay that allows for rapid identification of these genetically and antigenically distinct influenza B viruses is beneficial to understanding how the prevalence of these viruses varies in different geographic regions of the world and will provide data that could guide relevant vaccine strain selection.

Here we describe a real-time RT-PCR (rRT-PCR)-based assay for detection and discrimination of influenza B/VIC lineage deletion variants (Vic deletion assay). This assay is currently the only available method, to our knowledge, that can distinguish these B/VIC genetic groups, outside of a pyrosequencing technique and a conventional RT-PCR method, both recently published [11,12]. 

## Methods

### Influenza viruses, antigenic analysis and RNA extraction

Influenza viruses tested in this study were grown to high titre in either Madin–Darby canine kidney (MDCK) cells or embryonated chicken eggs (ECE) [13]. Infectious virus in culture supernatants or allantoic fluids was measured by 50% tissue culture infectious dose (TCID_50_/mL) or 50% egg-infectious dose (EID_50_ /mL), respectively [14]. Influenza B virus isolates used to evaluate analytical performance included B/Maryland/15/2016 and B/Colorado/6/2017 (V1A-2DEL), B/Hong Kong/269/2017 and B/Washington/02/2019 (V1A-3DEL) as well as no-deletion viruses B/Florida/103/2016 and B/Brisbane/60/2008 (V1A). We also included YAM lineage viruses represented by B/Phuket/3073/2013 and B/Texas/81/2016.

Influenza B virus lineages were confirmed using antigenic characterisation by HI assay on MDCK-propagated virus isolates and genetic sequence analysis. The antigenic characteristics of virus isolates were determined by HI tests using post-infection ferret sera raised against representative human influenza B viruses. The HI test was performed as described previously [15].

Viral RNA was extracted from 100 µL of supernatant or allantoic fluid and eluted into 100 µL of RNA elution buffer using a MagNA Pure Compact RNA isolation kit on a MagNA Pure Compact instrument (Roche Applied Science) [16].

### Primers and probes for the B/Victoria lineage deletion detection assay 

Oligonucleotide primers and probes for the Vic deletion assay were designed based on available nucleotide sequence data from the GenBank database of the National Center for Biotechnology Information (NCBI) and the Global Initiative on Sharing All Influenza Data (GISAID).

The Vic deletion assay includes a single set of conserved amplification primers and three deletion-specific dual-labelled hydrolysis probes, including VIC 2_Del, Vic 3_Del and Vic No_Del probes. The three probes, targeted to the deletion region of the HA gene of influenza B viruses, were designed to specifically detect and differentiate V1A-2DEL, V1A-3DEL and the V1A genetic group (no-deletion) viruses (B/VIC No_Del), respectively (Table 1). The Vic 2_Del and Vic No_Del probes were designed using Blackhole Quencher(BHQ)*plus* dual-labelled hydrolysis probes (BHQplus) that were labelled at the 5'-end with the reporter molecule 6-carboxyfluorescein (FAM) or Hex and CAL Fluor Red 610 and with BHQ1 (FAM or Hex) or BHQ2 (CAL Fluor Red 610) at the 3’-end. The Vic 3_Del probe was labelled at the 5'-end with the reporter molecule 6-carboxyfluorescein (FAM) and BHQ1 at the 3’-end, and included a triplet of locked nucleic acids (LNA) [17,18] that centred on the mismatch bases from V1A-2DEL and V1A viruses.

**Table 1:** Primer and probe sequences for the Centers for Disease Control and Prevention influenza B/Victoria lineage deletion detection assay

Primer/probe	Sequence 5'-3'	Nucleotide position^a^
Forward primer	GAT TYT TYG CAA TGG CTT G	497–518
Vic 2_Del probe^b^	CGT CCC AGA CAA AAA	522–542
Vic 3_Del probe^c^	TCC CAA AAA AC **AAA** ^d^ACA	524–544
Vic No_Del probe^b^	TTG TCG TTT GGG AC	539–523
Reverse primer	CCT TCT GTA CAA AYG TAT GGT ACT TC	596–571

HA: haemagglutinin; Vic: Victoria.

^a^ Nucleotide positions are according to HA gene of the B/Brisbane/60/2008(CY073893) strain.

^b^ The BHQPlus probe has FAM at the 5' end, BHQ1 at the 3' end and employs C-5 propynyl-dC (pdC) for dC and C-5 propynyl-dU (pdU) for dT substitutions (Glen Research Corporation, Virginia, US).

^c^ The BHQ and LNA probes have FAM at the 5'end, BHQ1 at the 3'end.

^d^ A triplet of locked nucleic acid residues.

We further evaluated primer and probe sequence specificity by sequence analysis of 51,759 gene segments available in the NCBI or GISAID databases. Primers were designed to have annealing temperatures of ca 60 °C and probes were designed to have annealing temperatures of ca 68 °C using PrimerExpress 3.0 software (Applied Biosystems, Foster City, US). Primers were synthesised by the Biotechnology Core Facility at the CDC.

### rRT-PCR reaction conditions

Reaction conditions for rRT-PCR were based upon the US Food and Drug Administration (FDA)-approved CDC Human Influenza Virus Real-Time RT-PCR Diagnostic Panel (Flu rRT-PCR Dx Panel) [16,19,20]. PCR reaction parameters of the Vic deletion assay were optimised using Invitrogen SuperScript III Platinum One-Step quantitative RT-PCR (qRT-PCR) (Single-plex) Kits (catalog no. 11732088, Life Technologies, Grand Island, US) and TaqPath qPCR Multiplex Master Mix (Triplex) kits (catalog no. A28526, Life Technologies) on the Applied Biosystems (AB) 7500 Fast Dx Real-Time PCR instrument. All rRT-PCR reactions performed had a total reaction volume of 25 µL with primer and probe reaction concentrations at 0.8 µM and 0.2 µM, respectively. Thermocycling rRT-PCR conditions were as follows: 50 °C for 30 min, *Taq* activation at 95 °C for 2 min and 45 cycles of 95 °C for 15 s and 55 °C for 30 s. All analytical performance data were collected on an ABI 7500 Fast Dx Real-time PCR instrument. Increases in fluorescent signal were registered during the annealing step of the reaction. All data were analysed with the sequence detector software (SDS) v1.4.1 (Life Technologies).

### Analytical sensitivity and specificity

In order to demonstrate rRT-PCR performance of the VIC deletion assay, five B/VIC lineage viruses and two B/YAM lineage viruses from recent circulating genetic groups, including the WHO-recommended influenza B vaccine viruses B/Colorado/6/2017 (V1A-2DEL), B/Brisbane/60/2008 (V1A) and B/Phuket/3073/2013 (B/Yam) were selected for assay evaluation (Figure). Analytical sensitivity of the Vic 2_Del and Vic No_Del assays was determined using two V1A-2DEL and V1A viruses. The Vic 3_Del assay was evaluated by using the V1A-3DEL virus B/Hong Kong/269/2017 and B/Washington/02/2019. The sensitivity of the three assays was evaluated with the commercially available Invitrogen SuperScript III Platinum One-Step qRT-PCR kit.

The analytical sensitivity and specificity of the Vic deletion assay were evaluated by comparison with the performance of the universal influenza B assay (InfB) from the CDC Flu rRT-PCR Dx Panel. The InfB assay is designed for detection of the nonstructural (NS) gene in all influenza B viruses by targeting highly conserved regions of the NS gene. A quantified synthetic RNA material (Armored RNA Quant CDC-9, AsuraGen, Inc., Austin, US) containing sequences from the NS gene region derived from B/Colorado/06/2017 (EPI 969380) was tested in triplicate to determine the minimum RNA copy number detectable by the InfB assay.

We further assessed the analytical specificity using these five B/VIC lineage deletion viruses and two B/YAM lineage viruses, 24 seasonal influenza A and two avian influenza A viruses.

### Assay performance on clinical specimens

To demonstrate the performance of the Vic deletion assay against clinical specimens, we tested 167 clinical specimens, including 67 B/VIC-positive and 30 B/YAM-positive to demonstrate the assay’s clinical sensitivity, as well as 30 seasonal influenza A-positive samples and 30 influenza-negative samples as determined by the CDC rRT-PCR Flu Panel to demonstrate the assay’s clinical specificity.

### Ethical statement

The specimens evaluated were collected in the course of public health surveillance and human subjects’ approval was not required for this project.

## Results

### Antigenic analysis

Influenza B viruses from the V1A, V1A-2DEL, V1A-3DEL and Yam lineages were antigenically characterised in HI tests using post-infection ferret sera raised against representative human influenza B viruses. The HI assays demonstrated that both the V1A-2DEL and the V1A-3DEL variant influenza B viruses were antigenically distinct from each other and from the no-deletion B/VIC virus (V1A), as the viruses from each of the genetic groups were not well inhibited by antiserum raised against the other genetic groups (reductions in HI titres of 4–32-fold compared with the homologous HI titres) (Table 2).

**Table 2:** Haemagglutination inhibition reactions of influenza B viruses

Influenza B virus strain	Ferret antiserum				
	V1A	V1A-2DEL	V1A-3DEL		YAM
	B/Brisbane/60/200	B/Maryland/15/2016	B/Hong Kong/286/2017	B/Washington/02/2019	B/Phuket/3073/2013
V1A					
**B/Brisbane/60/2008**	**2,560**	10	40	40	< 10
V1A-2DEL					
**B/Maryland/15/2016**	80	**160**	10	20	< 10
**B/Colorado/06/2017**	80	160	10	10	< 10
V1A-3DEL					
B/Hong Kong/286/2017	320	40	**320**	80	< 10
**B/Hong Kong/269/2017**	80	20	160	40	< 10
**B/Washington/02/2019**	40	40	40	**320**	< 10
Yamagata					
**B/Phuket/3073/2013**	< 10	< 10	< 10	< 10	**640**

Viruses used in this study are shown in bold and numbers in bold indicate homologous haemagglutination inhibition titres.

### Real-time RT-PCR assay establishment

The Vic deletion assay was developed using the ABI 7500 Fast Dx Real-time PCR system for the qualitative detection and characterisation of B/VIC virus RNA in respiratory specimens from patients presenting with influenza-like illness (ILI).

We designed three influenza B/VIC genetic group-specific probes to target the deletion region of aa position 162–164 (corresponding to nt position 529–537) of the HA gene in influenza B viruses (Table 1). The sequence surrounding the deletion contains multiple adenines and repeating sequences, creating a challenge for designing probes for this region. We found that unmodified RT-PCR probes were unsuccessful in targeting the deletion region owing to challenges in the surrounding sequence patterns (data not shown). BHQplus probes contain stabilising chemistry that allows a probe oligonucleotide to be a shorter length which more easily targets regions with challenging sequence patterns [21]. We used BHQplus chemistry to successfully design probes to the V1A and V1A-2DEL influenza B viruses (Table 1). When designing a probe for detection of the V1A-3DEL viruses, we attempted both a BHQplus design and a minor groove binding (MGB) probe for increased sequence specificity, but neither of these probe modifications were successful (data not shown). The LNA have been shown to increase stability and sequence mismatch detection [17,18], and we found that using a triplet of LNA centred on the mismatch allowed for successful detection of the V1A-3DEL viruses.

### Analytical sensitivity

Analytical performance studies, evaluated by testing 10-fold serial dilutions of RNA extracted from recently circulating influenza B viruses, demonstrated that the sensitivity of the VIC 2_Del, VIC 3_Del and VIC No_Del assays was comparable to the InfB assay of the CDC Flu rRT-PCR Dx Panel.

Analytical sensitivity of the Vic deletion assay is shown in Table 3 and Supplementary Table S2. Three quantified B/VIC viruses B/Maryland/15/2016 (V1A-2DEL), B/Hong Kong/269/2017 (V1A-3DEL) and B/Florida/103/2016 (V1A) were used to determine the limit of detection of the Vic 2_Del, Vic 3_Del and Vic No_Del assays, respectively. The results demonstrated that the sensitivity of Vic 2_Del and Vic No_Del assays were comparable to InfB assay, with < 3 cycle thresholds (Ct) difference between the InfB assay and the positive target of the Vic deletion assay. The Vic 3_Del assay was slightly less sensitive than the InfB assay, as the Ct value of Vic 3_Del probe was 3 Ct above the InfB target in two dilutions of B/Hong Kong/269/2017 (Table 3).

**Table 3:** Assay limit of detection determined using quantified influenza B/Victoria lineage viruses in comparison with the quantified synthetic RNA (tested in triplicate)

RNA copies^a^	Ct Value^b^	Virus titre (TCID_50_/mL)	Ct Value^b^		Virus titre (EID_50_/mL)	Ct Value^b^		Virus titre (TCID_50_/mL)	Ct value^b^	
	InfB		InfB	VIC No_Del		InfB	VIC 2_Del		InfB	VIC 3_Del
**Armored RNA**		**B/Florida/103/2016**			**B/Maryland/15/2016**			**B/Washington/2/2019**		
5,000	26.38 ± 0.17	10 ^3.3^	26.23 ± 0.59	24.06 ± 0.09	10 ^4.5^	26.17 ± 0.32	24.53 ± 0.36	10 ^1.2^	27.64 ± 0.79	28.85 ± 1.65
500	29.90 ± 0.27	10 ^2.3^	30.53 ± 0.56	28.38 ± 0.09	10 ^3.5^	29.60 ± 0.91	28.70 ± 0.14	10 ^0.2^	31.70 ± 0.77	34.41 ± 1.75
50	33.14 ± 0.22	10 ^1.3^	34.77 ± 0.98	31.80 ± 0.45	10 ^2.5^	35.19 ± 1.06	32.60 ± 0.26	**10** ^−^ **^0.2^**	**33.45 ± 0.38**	**37.31 ± 0.15**
**5**	**35.12 ± 0.92**	**10 ^0.3^**	**35.60 ± 1.16**	**35.35 ± 0.17**	**10 ^1.5^**	**36.14 ± 0.32**	**37.22 ± 0.89**			

Ct: cycle threshold; EID: egg-infectious dose; InfB: universal influenza B assay; TCID: tissue culture-infectious dose; VIC: Victoria.

^a^ The RNA material (Armored RNA Quant CDC-9, received from AsuraGen,Inc.) included the highly conserved InfB primer/probe sequence of the NS gene region, derived from the influenza B/Victoria lineage virus B/Colorado/06/2017(EPI 969380). The reference virus sequences in this region were 100% identical to Armored RNA Quant CDC-9.

^b^ Mean ± standard deviation.

The virus titres and RNA copies at the level of the assay’s limit of detection are highlighted in bold.

The limits of detection of Vic 2_Del, Vic 3_Del and Vic No_Del assays were 10^1.5^, 10^−0.2^ and 10^0.3^ ID_50_/mL, respectively (10^−0.2~1.5^ ID_50_/mL). This correlates to 10^−2.5~ −0.8^ ID_50_ per reaction (5.0 µL/reaction). To understand how many copies of RNA per reaction the deletion assay will detect, we first used Armored RNA Quant CDC-9-containing sequences from the NS gene region derived from B/Colorado/06/2017 to evaluate the InfB assay. We then matched rRT-PCR results of the serial dilution of virus titres to RNA copies based on comparable Ct values in the InfB assay. Given this comparison, we estimate that the Vic 2_Del and Vic No_Del probes will detect five RNA copies per reaction and the Vic 3_Del probe will detect ca 50 RNA copies per reaction. Therefore, the sensitivity of the Vic deletion assay was ca 5–50 RNA copies per reaction (Table 3). Two additional V1A viruses, B/Colorado/06/2017 (V1A-2DEL) and B/Brisbane/60/2008 (V1A), were used to evaluate the Vic deletion assay within the lower virus titres, and similar sensitivity was observed (Supplementary Table S2).

We also developed a triplex rRT-PCR assay with one pair of conserved primers with FAM-labelled Vic 3_Del probe, Hex-labelled Vic 2_Del probe and CAL Fluor Red 610-labelled Vic No_Del probe. This triplex rRT-PCR Vic deletion assay showed the same level of sensitivity and specificity as the singleplex assays in detecting and discriminating the B/VIC deletion variant viruses (Supplementary Table S2).

### Analytical specificity (exclusivity)

We evaluated analytical exclusivity with high-titre influenza B VIC and YAM lineage viruses (Table 4). We did not observe any cross-reactivity when V1A viruses were tested with the VIC 2_Del and VIC 3_Del assays. Specificity was also demonstrated in V1A-3DEL viruses tested with the VIC 2_Del assay and vice versa. We did not observe cross-reactivity from the VIC No_Del assay when used to test V1A-2DEL and V1A-3DEL viruses. Likewise, the three assays did not react with the B/YAM lineage viruses tested (Table 4).

**Table 4:** Analytical specificity (exclusivity) testing with influenza B Victoria and Yamagata lineage viruses

Influenza B virus	Lineage	Genetic group	Infectious titre (ID_50_/mL)	Ct value			
				InfB	VIC 2_Del	Vic 3_Del	VIC No_Del
B/Maryland/15/2016	VIC	V1A 2-DEL	10^8.5 a^	9.21	9.52	Negative	Negative
B/Colorado/6/2017	VIC	V1A 2-DEL	10^9.4 a^	10.14	10.18	Negative	Negative
B/Hong Kong/269/2017	VIC	V1A 3-DEL	10^4.2 b^	14.92	Negative	16.01	Negative
B/Washington/02/2019	VIC	V1A 3-DEL	10^8.2 a^	15.61	Negative	17.29	Negative
B/Florida/103/2016	VIC	V1A	10^6.3 b^	10.96	Negative	Negative	11.80
B/Brisbane/60/2008	VIC	V1A	10^7.9 a^	15.67	Negative	Negative	16.27
B/Phuket/3073/2013	YAM	Not applicable	10^7.9 a^	17.54	Negative	Negative	Negative
B/Texas/81/2016	YAM	Not applicable	10^8.3 a^	13.40	Negative	Negative	Negative

Ct: cycle threshold; EID: egg-infectious dose; InfB: universal influenza B assay; TCID: tissue culture-infectious dose; VIC: Victoria; YAM: Yamagata.

^a^ Data represent EID_50_/mL.

^b^ Data represent TCID_50_/mL.

In order to demonstrate the absence of cross-reactivity with influenza A viruses subtypes, we performed exclusivity testing by examining 12 isolates each of contemporary seasonal influenza A(H3N2) and A(H1N1)pdm09 viruses as well as the benchmark highly pathogenic avian influenza (HPAI) A(H5N1) strain, A/Vietnam/1203/2004, and Asian lineage avian influenza A(H7N9) virus, A/Anhui/01/2013. All influenza A viruses were negative in all three assays (Supplementary Table S3).

### Clinical performance of influenza B/Victoria lineage deletion detection assay

To evaluate the clinical performance of the Vic deletion assay, we tested the assay on clinical specimens received at the CDC during influenza surveillance. The Vic deletion assay was successful in classifying the 77 B/VIC clinical specimens into their respective genetic groups (30 V1A-like, 27 V1A-2DEL-like and 20 V1A-3DEL-like) and, the rRT-PCR results were confirmed by sequencing (Table 5). All 30 influenza B/YAM, 15 each of influenza A(H1N1)pdm09 and A(H3N2), as well as 30 influenza A and B-negative samples tested negative for all B/VIC deletion assay targets (Table 6).

**Table 5:** Clinical performance of the influenza B/Victoria lineage deletion detection assay: detection of B/Victoria lineage specimens compared with sequencing results

B/VIC deletion assay positive	Sequencing results^a^		
	V1A	V1A-2DEL	V1A-3DEL
Vic No_Del (n = 27 positive samples)	27	0	0
Vic 2_Del (n = 30 positive samples)	0	30	0
Vic 3_Del (n = 20 positive samples)	0	0	20

Vic: Victoria.

^a^ Genetic groups were determined by genetic analysis.

**Table 6:** Clinical performance of the influenza B/Victoria lineage deletion detection assay: detection of other subtype/lineage influenza specimens compared with the CDC Human Influenza Virus Real-Time RT-PCR Diagnostic Panel (CDC Flu rRT-PCR Dx Panel)

CDC Flu rRT-PCR Dx Panel result	B/VIC deletion assay^a^	
	Positive	Negative
Influenza A (H1N1)pdm09 and (H3N2)	0	30 (15 + 15)
Influenza A and B-negative	0	30
Influenza B/YAM	0	30

CDC: United States Centers for Disease Control and Prevention; Vic: Victoria; YAM: Yamagata.

^a^ Including Vic No_Del, Vic 2_Del and Vic 3_Del targets.

## Discussion

The Vic deletion assay presented here is intended for the qualitative detection of the influenza B/VIC lineage HA gene deletion variant viruses using rRT-PCR technology. The analytical and clinical performance of the Vic deletion assay in either the singleplex or triplex configuration demonstrates that the assay is highly efficient, sensitive and comparable to the gold standard CDC rRT-PCR Flu Panel InfB assay [20]. We have further shown that the primers and probes of the VIC deletion assay are specific to V1A, V1A-2DEL and V1A-3DEL viruses and do not cross-react with B/YAM viruses, seasonal influenza A, or avian influenza A viruses including HPAI A(H5N1) and Asian lineage A(H7N9) influenza viruses.

The key genetic distinction between V1A, V1A-2DEL, and V1A-3DEL viruses is within the same nucleic acid region, presenting a challenge for rRT-PCR probe design. We used two chemical modifications in probe design, LNA and BHQplus chemistry, which allowed for greater stability and specificity. Including LNA in real-time PCR probes improves detection of mismatches significantly [16,17]. The Vic 3_Del probe was designed and optimised using a triplet LNA approach, as probes labelled with a triplet of LNA residues centred on the mismatch provide greater discriminatory power than probes with a single LNA modification [17,18]. The probes for V1A and V1A-2DEL were designed using BHQplus chemistry, allowing for stabilisation and enhanced mismatch detection with an overall shorter probe length [21]. We found that unmodified probes were not sufficient for this assay.

Sequence alignments using HA gene sequences from 2010 to 2019 demonstrated the conserved primers to be stable with no conserved genetic changes observed (data not shown). Although the areas chosen for the conserved amplification primers and the deletion type-specific probes are currently stable, genetic changes following rapid virus evolution and the variable nature of RNA viruses may require periodic updates of the Vic deletion assay primer and probe sequences.

The number and percentage of circulating V1A-2DEL and V1A-3DEL viruses has increased significantly since initial identification [22], making it critical to have a simplified and sensitive assay capable of detecting these genetic variants during influenza surveillance. The Vic deletion rRT-PCR assay is the most sensitive method currently available that can distinguish these influenza B genetic subgroups. One limitation of our assay is that it cannot distinguish between the two antigenically distinct groups within the V1A-3DEL viruses (V1A.2 and V1A.3), but it does detect V1A-3DEL viruses, regardless of antigenicity. Recently, a pyrosequencing method was published that can also be used to distinguish these influenza B genetic variants [11]. However, the pyrosequencing method requires costly instruments that are not common to diagnostic laboratories, and rRT-PCR is more sensitive than sequencing-based methods [23,24]. A conventional RT-PCR assay has also recently been developed to distinguish these influenza B genetic subgroups, but conventional RT-PCR is less sensitive and more time-consuming than an rRT-PCR assay [12]. 

## Conclusion

Given its sensitivity and specificity, the rapidity of results, the fact that the Vic deletion assay can be run on equipment that is readily available in most diagnostic laboratories and the fact that the assay can be multiplexed for testing efficiency and cost-savings, this is a suitable assay for influenza surveillance. Combining the CDC Vic deletion assay with the CDC Flu rRT-PCR Dx Panel Influenza B Lineage Genotyping Kit will allow for rapid identification of the distinct influenza B viral genetic groups B/YAM, B/VIC V1A, V1A-2DEL and V1A-3DEL [19]. This will further our understanding of the health impact of each of these antigenically and genetically distinct viruses in different global regions, which in turn will contribute to the most relevant vaccine virus recommendations moving forward into future influenza seasons.

## Supplementary Data

35000 Supplement
