# Erratum for Euro Surveill. 2020;25(41)

**DOI:** 10.2807/1560-7917.ES.2020.25.42.201022e

**Published:** 2020-10-22

**Authors:** Eurosurveillance editorial team

**Affiliations:** 1 European Centre for Disease Prevention and Control (ECDC), Stockholm, Sweden

In the pdf version of the article "Detection and discrimination of influenza B Victoria lineage deletion variant viruses by real-time RT-PCR" by Shu et al, published on 15 October 2020, the ID_50_ values in Tables 3 and 4 were incorrectly printed without superscripts. This mistake has not affected the online html version of the article. The pdf was corrected on 22 October. We apologise for the error.

