# Supplementary material for: Detection and discrimination of influenza B Victoria lineage deletion variant viruses by real-time RT-PCR
Source: Euro Surveill. 2020 Oct 15;25(41):1900652. doi: 10.2807/1560-7917.ES.2020.25.41.1900652 (PMC7565853; doi:10.2807/1560-7917.ES.2020.25.41.1900652)
Supplement: Supplement [file 19-00652_BARNES_Supplement.pdf]

Supplementary Material

This supplementary material is hosted by *Eurosurveillance* as supporting information alongside the article, “Detection and discrimination of influenza B Victoria lineage deletion variant viruses by real-time RT-PCR” on behalf of the authors, who remain responsible for the accuracy and appropriateness of the content. The same standards for ethics, copyright, attributions and permissions as for the article apply. Supplements are not edited by *Eurosurveillance* and the journal is not responsible for the maintenance of any links or email addresses provided therein.

Table S1. The HA gene sequences of Influenza B viruses analyzed in figure 1

Influenza B virus stain	Lineage	Genetic groups	GISAID Accession No
B/Louisiana/01/2019	VIC	V1A-3DEL	EPI1357124
B/Washington/02/2019	VIC	V1A-3DEL	EPI1395080
B/Sichuan-Qingyang/12081/2018	VIC	V1A-3DEL	EPI1351221
B/Sichuan-Gaoxin/51/2019	VIC	V1A-3DEL	EPI1351209
B/Manitoba/RV0457/2019	VIC	V1A-3DEL	EPI1358366
B/Texas/7671/2018	VIC	V1A-3DEL	EPI1538372
B/Sweden/61/2018	VIC	V1A-3DEL	EPI1338321
B/Ghana/4134/2018	VIC	V1A-3DEL	EPI1354861
B/Paris/2659/2018	VIC	V1A-3DEL	EPI1338503
B/Sierra_Leone/184/2018	VIC	V1A-3DEL	EPI1347779
B/Mexico/209/2017	VIC	V1A-3DEL	EPI980747
B/Norway/4183/2018	VIC	V1A-3DEL	EPI1371139
B/Bangkok/129/2018	VIC	V1A-3DEL	EPI1362553
B/Hong_Kong/269/2017	VIC	V1A-3DEL	EPI1106050
B/Laos/F1664/2017	VIC	V1A-3DEL	EPI1147409
B/Newcastle/63/2018	VIC	V1A-2DEL	EPI1359291
B/New_Mexico/16/2018	VIC	V1A-2DEL	EPI1348272
B/Jiangxi-Donghu/321/2018	VIC	V1A-2DEL	EPI1351182
B/Manitoba/RV0309/2019	VIC	V1A-2DEL	EPI1355486
B/Montana/36/2018	VIC	V1A-2DEL	EPI1357171
B/New_Brunswick/RV0097/2019	VIC	V1A-2DEL	EPI1341257
B/Peru/9217/2017	VIC	V1A-2DEL	EPI1051862
B/Illinois/22/2016	VIC	V1A-2DEL	EPI924523
B/Maryland/15/2016	VIC	V1A-2DEL	EPI1255272

B/El_Salvador/763/2018	VIC	V1A-2DEL	EPI1343445
B/Colorado/06/2017	VIC	V1A-2DEL	EPI1011552
B/Norway/2409/2017	VIC	V1A-2DEL	EPI1138013
B/Hong_Kong/1412/2018	VIC	V1A-2DEL	EPI1393949
B/Sri_Lanka/24/2018	VIC	V1A-2DEL	EPI1359327
B/Oman/6001/2018	VIC	V1A	EPI1328991
B/Uruguay/138/2017	VIC	V1A	EPI1034180
B/Malaysia/RP3746/2018	VIC	V1A	EPI1359432
B/Brisbane/60/2008	VIC	V1A	EPI244699
B/South_Australia/81/2012	VIC	V1A	EPI566499
B/Alaska/15/2017	VIC	V1A	EPI1053446
B/Florida/103/2016	VIC	V1A	EPI892450
B/New_Hampshire/01/2018	YAM		EPI1167112
B/Fujian-Tongan/1907/2016	YAM		EPI1059771
B/Phuket/3073/2013	YAM		EPI544264
B/Texas/81/2016	YAM		EPI858300

Table S2. Analytical sensitivity of the B/Victoria Lineage Deletion Detection assay single- and multi-plex assays: Vic 2_Del assay against B/Vic V1A-2DEL viruses (S2a), Vic 3_Del assay against V1A-3DEL virus (S2b) and Vic No_Del assay against B/Vic V1A-the other B/Vic viruses (S2c) (N=3)

S2a

Influenza B V1A-2DEL viruses (EID_{50}/mL)	Ct Value (Mean±SD)		
	InfB	Vic 2_Del	
		Single (FAM)	Triplex (Hex)
B/Maryland/15/2016			
$10^{4.5}$	26.17±0.32	24.53±0.36	26.13±0.04
$10^{3.5}$	29.60±0.91	28.70±0.14	29.86±0.12
$10^{2.5}$	35.19±1.06	32.60±0.26	34.20±0.26
B/Colorado/06/2017			
$10^{4.4}$	29.48±0.43	27.95±0.23	29.26±0.21
$10^{3.4}$	33.54±0.28	32.38±010	33.57±0.09
$10^{2.4}$	37.13±0.97	35.84±1.31	36.28±0.03

S2b

Influenza B V1A-3DEL virus ($TCID_{50}$/mL)	Ct Value (Mean±SD)		
	InfB	Vic 3_Del	
		Single (FAM)	Triplex (FAM)
B/Hong Kong/269/2017			
$10^{2.2*}$	22.93±0.51	26.37±1.29	26.48±0.18
$10^{1.2}$	27.64±0.79	28.85±.65	30.12±0.15
$10^{0.2}$	31.70±0.77	34.41±1.75	33.63±0.35

S2c

Influenza B V1A viruses (ID_{50}/mL)	Ct Value (Mean±SD)		
	InfB	Vic No_Del	
		Single (FAM)	Triplex (Cal610)
B/Florida/103/2016			
$10^{3.3a}$	26.23±0.59	24.06±0.09	25.10±0.13
$10^{2.3}$	30.53±0.56	28.38±0.09	29.94±0.60
$10^{1.3}$	34.77±0.98	31.80±0.45	33.47±0.36
B/Brisbane/60/2008			
$10^{4.9b}$	29.47±0.55	26.65±0.28	28.38±0.12
$10^{3.9}$	34.42±0.97	31.14±0.06	32.70±0.37
$10^{2.9}$	36.79±0.19	34.54±0.27	37.29±0.26

[a]Data represent TCID50/mL; [b]Data represent EID50/mL

Table S3. Analytical Specificity (Exclusivity) Testing with Influenza A Viruses (N=1)

Influenza A virus	Subtype	Infectious Titer (ID50/ml)	rRT-PCR result		
			VIC 2_Del	VIC 3_Del	VIC No_Del
A/California/07/2009	H1N1pdm09	$10^{7.5a}$	-	-	-
A/Brisbane/10/2010	H1N1pdm09	$10^{7.2a}$	-	-	-
A/Christ Church/16/2010	H1N1pdm09	$10^{8.9a}$	-	-	-
A/Minnesota/03/2011	H1N1pdm09	$10^{8.9a}$	-	-	-
A/Voronezh/1/2011	H1N1pdm09	$10^{8.4a}$	-	-	-
A/Bangladesh/2021/2012	H1N1pdm09	$10^{8.1a}$	-	-	-
A/Maryland/13/2012	H1N1pdm09	$10^{5.0b}$	-	-	-
A/Colorado/14/2012	H1N1pdm09	$10^{5.1b}$	-	-	-
A/Michigan/45/2015	H1N1pdm09	$10^{7.2a}$	-	-	-
A/West Virginia/01/2016	H1N1pdm09	$10^{5.4b}$	-	-	-
A/Illinois/20/2018	H1N1pdm09	$10^{6.8b}$	-	-	-
A/Brisbane/02/2018	H1N1pdm09	$10^{6.9b}$	-	-	-
A/Hawaii/08/2006	H3N2	$10^{7.8b}$	-	-	-
A/Uruguay/716/2007	H3N2	$10^{8.2a}$	-	-	-
A/Afghanistan/2903/2008	H3N2	$10^{5.0b}$	-	-	-
A/Texas/50/2012	H3N2	$10^{6.2b}$	-	-	-
A/Stockholm/06/2014	H3N2	$10^{8.1a}$	-	-	-
A/Canberra/82/2014	H3N2	$10^{6.9a}$	-	-	-
A/Hong Kong/4801/2014	H3N2	$10^{8.4a}$	-	-	-
A/Texas/88/2016	H3N2	$10^{6.5b}$			
A/Idaho/33/2016	H3N2	$10^{7.9a}$	-	-	-
A/Singapore/INFIMH-16-0019/2016	H3N2	$10^{7.2a}$	-	-	-
A/Texas/88/2016	H3N2	$10^{7.5a}$	-	-	-
A/Switzerland/8060/2017	H3N2	$10^{8.2a}$	-	-	-
A/Vietnam/1203/2014	H5N1	$10^{6.8a}$	-	-	-
A/Anhui/1/2013	H7N9	$10^{8.9a}$	-	-	-

[a]EID50/ml; [b]TCID50/ml
